# Directed evolution of bright mutants of an oxygen-independent flavin-binding fluorescent protein from *Pseudomonas putida*

**DOI:** 10.1186/1754-1611-6-20

**Published:** 2012-10-24

**Authors:** Arnab Mukherjee, Kevin B Weyant, Joshua Walker, Charles M Schroeder

**Affiliations:** 1 Department of Chemical and Biomolecular Engineering, University of Illinois at Urbana-Champaign, Urbana, IL 61801, USA; 2 Center for Biophysics and Computational Biology, University of Illinois at Urbana-Champaign, Urbana, IL 61801, USA; 3 Department of Materials Science and Engineering, University of Illinois at Urbana-Champaign, Urbana, IL 61801, USA

**Keywords:** Flavin-binding fluorescent proteins, Directed evolution, Site saturation mutagenesis

## Abstract

**Background:**

Fluorescent reporter proteins have revolutionized our understanding of cellular bioprocesses by enabling live cell imaging with exquisite spatio-temporal resolution. Existing fluorescent proteins are predominantly based on the green fluorescent protein (GFP) and related analogs. However, GFP-family proteins strictly require molecular oxygen for maturation of fluorescence, which precludes their application for investigating biological processes in low-oxygen environments. A new class of oxygen-independent fluorescent reporter proteins was recently reported based on flavin-binding photosensors from *Bacillus subtilis* and *Pseudomonas putida*. However, flavin-binding fluorescent proteins show very limited brightness, which restricts their utility as biological imaging probes.

**Results:**

In this work, we report the discovery of bright mutants of a flavin-binding fluorescent protein from *P. putida* using directed evolution by site saturation mutagenesis. We discovered two mutations at a chromophore-proximal amino acid (F37S and F37T) that confer a twofold enhancement in brightness relative to the wild type fluorescent protein through improvements in quantum yield and holoprotein fraction. In addition, we observed that substitution with other aromatic amino acids at this residue (F37Y and F37W) severely diminishes fluorescence emission. Therefore, we identify F37 as a key amino acid residue in determining fluorescence.

**Conclusions:**

To increase the scope and utility of flavin-binding fluorescent proteins as practical fluorescent reporters, there is a strong need for improved variants of the wild type protein. Our work reports on the application of site saturation mutagenesis to isolate brighter variants of a flavin-binding fluorescent protein, which is a first-of-its-kind approach. Overall, we anticipate that the improved variants will find pervasive use as fluorescent reporters for biological studies in low-oxygen environments.

## Background

Green fluorescent protein (GFP) and related analogs have been extensively engineered by directed evolution [1-4] to evolve fluorescent reporters with faster maturation times [5,6], enhanced brightness [7-9], improved photostability [10], a wide range of emission wavelengths [11,12], enhanced thermal tolerance [13], and improved efficiency of Förster resonance energy transfer (FRET) [14]. However, the available palette of GFP-based fluorescent proteins is limited by a dependence on molecular oxygen, which mediates oxidation of a cyclic tripeptide chromophore that is strictly required for fluorescence [15,16]. In this way, GFP-family reporters require oxygen for fluorescence and do not fluoresce in anaerobic environments [17-20].

Low oxygen environments are frequently encountered in a broad range of biomedical and industrial bioprocesses, including bioremediation and fermentation platforms for the production of high value reduced biomolecules (*e.g.*, biofuels), hypoxic tissue environments that promote tumorigenesis, microbial pathogenesis and biofilm development, and in biotechnology applications based on obligate anaerobes. Consequently, there is a pressing need to develop a new class of reporter proteins that is capable of fluorescence in microaerobic and anaerobic environments. Recently, two flavin-binding photosensory proteins were isolated from *P. putida* and *B. subtilis* and were shown to fluoresce upon heterologous expression in *E. coli*[21]. In a separate study, a flavin-binding photosensory protein based on a blue light photoreceptor (phototropin) from *A. thaliana* was isolated and used to track the development of viral lesions in tobacco mosaic virus-infected *N. tabacum* leaves [22]. Importantly, flavin-binding fluorescent proteins (FbFPs) were observed to efficiently fluoresce when expressed in anaerobically cultured *E. coli* cells under identical conditions in which the GFP-family yellow fluorescent protein (YFP) was rendered nonfluorescent [21]. FbFPs comprise a conserved light, oxygen, or voltage-sensing (LOV) domain core, which is widely employed by plant phototropins (blue light receptors) for phototaxis, stomatal opening of guard cells, chloroplast translocation, and a variety of light-driven regulatory responses [23-25]. FbFPs have a characteristic Per Arnt Sim (PAS) fold and employ a noncovalently bound flavin mononucleotide (FMN) cofactor as the light-sensing moiety [26]. Flavin-binding photosensors have been implicated in mediating diverse functions in prokaryotes, including regulation of stress response and virulence and mediating cell adhesion [27,28].

**Figure 1:**
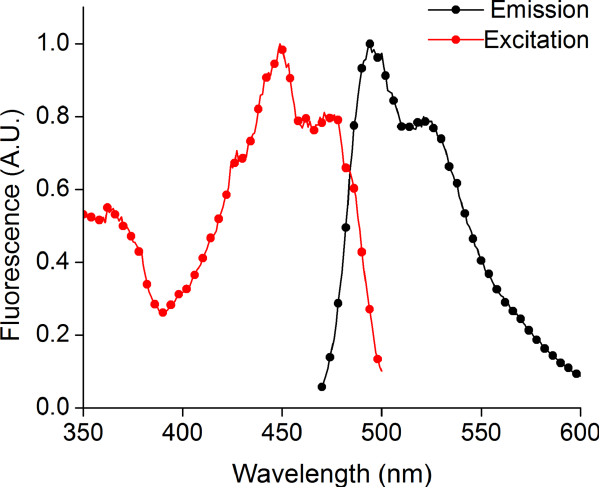
**Fluorescence excitation and emission spectra for purified wild type FbFP protein. **(a) Excitation spectrum of purified FbFP in Buffer E (25 mM sodium phosphate, 1 M sodium chloride, pH 7.4). Excitation spectrum was determined by monitoring fluorescence emission at 500 nm while scanning the excitation light between 350 to 480 nm. (b) Emission spectrum of purified FbFP in Buffer E. Emission spectrum was determined by monitoring fluorescence emission between 470 and 600 nm while exciting the sample at 450 nm.

Flavin-binding photosensors from *B. subtilis* and *P. putida* exhibit a complex photocycle in nature. Wild type photosensory proteins show an absorption maximum at 450 nm, but they are nonfluorescent upon excitation at this wavelength. Illumination with blue light converts the FMN chromophore into an excited singlet state, followed by fast conversion to an excited triplet state by intersystem crossing. The triplet state is characterized by a red-shifted absorption maximum at 660 nm and decays within 1–2 μs to a species with a blue-shifted absorption peak at 390 nm. The protein-cofactor species with blue-shifted absorption is thought to involve a covalent adduct between FMN and a nearby cysteine residue in the protein. The FMN-cysteine adduct slowly reverts (~seconds) to the ground state conformation, wherein the FMN molecule is noncovalently buried in the protein cavity [29-33]. Repeated photocycling between the ground state, the excited state, and the FMN-protein covalent adduct results in no net fluorescence emission. However, mutation of the cysteine residue to alanine has been shown to eliminate this photocycle. Mutant proteins incorporating a noncovalently associated FMN (via the cysteine to alanine mutation) show a broad fluorescence emission with a peak at 495 nm upon excitation with blue light at 450 nm [21,34] (Figure 1), thereby providing a functional flavin-binding fluorescent protein (FbFP) with oxygen-independent fluorescence emission.

FbFPs have been utilized in only a limited set of biological studies such as plasmid conjugation in *E. coli*[35]. Codon-optimized variants of the reporters have been shown to fluoresce when heterologously expressed in *Saccharomyces cerevisiae*, *Candida albicans*, *Bacteroides fragilis*, and *Porphyromonas gingivalis* in anaerobic conditions [36-38]. Under growth conditions that favor rapid bacterial growth, FbFPs exhibited superior performance compared to YFP as reporters of gene expression [39]. In this work, fluctuations in oxygen tension occurred in the growth medium as cells transitioned from an actively respiring growth phase to the stationary phase, thereby resulting in time-dependent fluorescence of YFP due to fluctuations in oxygen, whereas the FbFPs were observed to be insensitive to variable oxygen tension. In a recent study, translational fusions between O_2_-independent FbFP and O_2_-sensitive YFP were constructed to generate FRET-based biosensors for real-time monitoring of cellular oxygen concentrations in *E. coli*[40].

FbFPs are promising candidates to serve as a new class of fluorescent reporters in low-oxygen conditions. In addition to oxygen-independent fluorescence emission, FbFPs exhibit a relatively small size (≈150 amino acids), which is a desirable feature for generating fluorescently labeled fusion proteins with small imprints. However, versatile application of FbFPs as robust imaging probes is currently hindered by their limited brightness. Indeed, fluorescence emission from FbFPs is weak unless FbFPs are expressed at elevated levels using strong promoters [35-40]. Based on the considerable promise of FbFPs as a new class of fluorescent reporter proteins, we aimed to evolve an FbFP for enhanced brightness and improved spectral properties. In this work, we evolved the FbFP isolated from *P. putida*, which has been shown to express well in both *E. coli* and the obligate anaerobe *Rhodobacter capsulatus*[21]. We isolated two mutants involving an FMN-proximal amino acid (F37S and F37T) that confer a twofold enhancement in brightness of fluorescence emission relative to the wild type protein. Based on biochemical characterization, we conjecture that the F37S and F37T mutations improve brightness by relieving fluorescence quenching stacking interactions in the wild type protein, thereby increasing quantum yield, and by enriching the fraction of FMN-bound fluorescent holoprotein in the evolved mutants. Overall, we anticipate that these improved spectral variants will be valuable for investigating biological processes in low-oxygen conditions.

## Results and discussion

### Homology modeling and design of site saturation mutagenesis

FbFP fluorescence is mediated by a buried FMN chromophore and the fluorescence emission of FMN is known to be sensitive to its microenvironment [41]. Therefore, we hypothesized that mutations within the chromophore-binding cavity could be used to enhance the fluorescence of wild type FbFPs by affecting the photophysical interactions between FMN and its neighboring amino acids. Therefore, we evolved FbFP using saturation mutagenesis of chromophore-proximal amino acids. Site saturation mutagenesis enables the incorporation all possible amino acid substitutions at selected target sites [42]. Using this approach, degenerate oligonucleotides are used to engineer amino acid substitutions into proteins without the need for rigorously defined structure-function relationships. Previously, site saturation mutagenesis has been used to engineer key improvements in the fucose hydrolyzing activity and thermostability of an *E. coli* beta-galactosidase and a *B. subtilis* lipase respectively, by mutating amino acids in the active site [43-45]. In recent work, saturation mutagenesis of amino acids proximal to the pyrrole chromophore of a bacterial phytochrome was employed by the Tsien lab to evolve an infrared emitting fluorescent protein [46].

**Figure 2:**
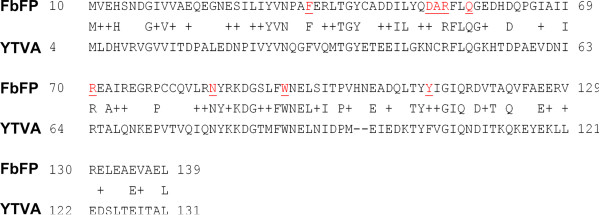
**Sequence alignment between FbFP and YTVA (*B. subtilis*). **Sequence alignment reveals close agreement between FbFP from *P. putida* and YTVA from *B. subtilis*, with 37% identical and 62% similar amino acids shared between the two proteins. In this way, the YTVA structure serves as an ideal template for molecular modeling of the FbFP. Amino acids selected for site saturation mutagenesis are indicated in red and underlined.

**Figure 3:**
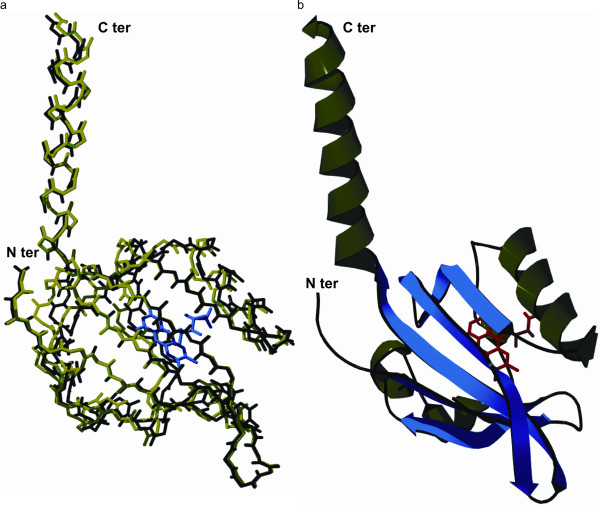
**Homology derived structure of FbFP. (a**) Alignment between the backbone Cα atoms of the YTVA template (green) and the FbFP model (black). The structures are well aligned, with a root mean squared deviation of 0.5 nm over 126 Cα atoms. The FMN cofactor is depicted in blue. (**b**) Ribbon model of homology derived three dimensional structure of FbFP. The buried FMN cofactor is indicated in red. Alpha helices and beta strands in the protein structure are represented in green and blue respectively. Images were generated by ray tracing with POV-Ray version 3.6.

In this work, we applied site saturation mutagenesis to FbFP to improve the spectral properties of the fluorescent protein. First, we modeled the structure of FbFP from *P. putida* based on the closely related LOV domain protein YTVA from *B. subtilis* (UniProt: O34627), which shares 37% identity and 62% similarity in primary sequence (Figure 2). The YTVA protein comprises a signal receiving LOV domain coupled to an effector STAS (sulfate transporter anti sigma factor antagonist) domain. YTVA is implicated in mediating light-driven stress regulatory response in *B. subtilis* by activating the stress responsive transcription factor σ^B^[28,47,48]. Homology modeling was implemented using Swiss PDB Viewer version 4.0. We determined a homology modeled structure using an energy minimization method, wherein the refined protein structure presented 96% of amino acids in allowed regions of the Ramachandran plot and showed a root mean squared deviation of 0.5 nm over 126 backbone carbon atoms (Figure 3).

**Figure 4:**
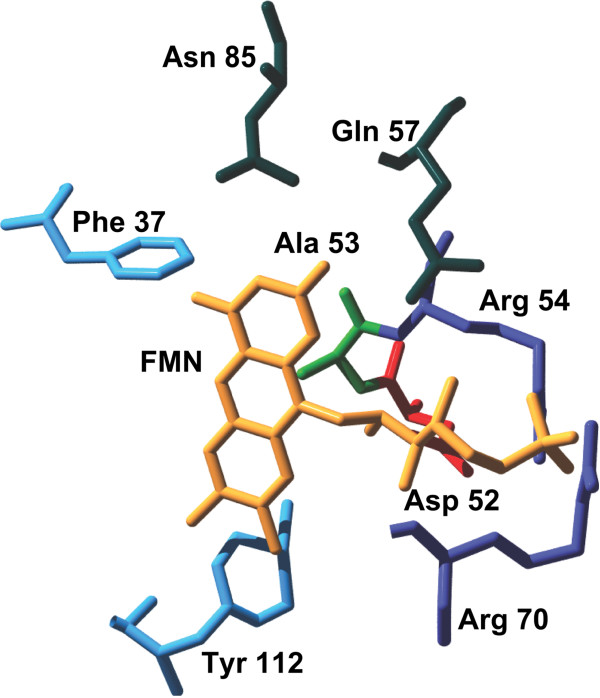
**Flavin-binding cavity in FbFP. **Amino acids upto a distance of 0.3 nm from FMN are shown. Also shown are A52 and F37 which are at a distance of 0.4 nm from FMN. Amino acids are colored according to their property (blue – aromatic; red - negatively charged; violet - positively charged; green – hydrophobic; deep green - polar). The W94 target lies outside the vicinity of the chromophore and does not appear in the structure.

We used the protein structure to select six amino acids up to a distance of 0.3 nm from the FMN chromophore, including D52, R54, R70, and Q57, which comprise a signature motif for the FMN-binding LOV domain [29], and nearby amino acids Y112 and N85 (Figure 4). In addition to the aforementioned six FMN-proximal amino acids, we also selected the following three amino acids for mutagenesis: 1) A53 (C53 in the wild type FbFP) located 0.4 nm from FMN, because the cysteine residue in the wild type protein forms a covalent adduct with FMN upon blue light excitation. 2) F37 residue located approximately 0.4 nm from the FMN ring, because the close proximity of an aromatic amino acid to the FMN chromophore suggests the possibility of π-bond stacking interactions between the phenyl ring and the isoalloxazine ring of FMN. 3) W94 residue, which lies farther away from the FMN chromophore, but has been implicated in mediating interdomain contacts in the FbFP [29]. A summary of oligonucleotides used for site saturation mutagenesis is shown in Table 1.

**Table 1:** Oligonucleotides used in this work

**No.**	**Primer**	**Sequence**
1	FbFP_ampl_fwd	GGATCCATGATCAACGCAAAACTCCTG
2	FbFP_ampl_rev	AAGCTTTCAGTGCTTGGCCTGGCC
3	FbFP_F37Xmut	CGTCAACCCGGCC**NNK**GAGCGCCTGACC
4	FbFP_D52Xmut	CGATATTCTCTATCAG**NNK**GCACGTTTTCTTCAGG
5	FbFP_A53Xmut	GACGATATTCTCTATCAGGAC**NNK**CGTTTTCTTCAGGGCGAGGAT
6	FbFP_R54Xmut	TTCTCTATCAGGACGCA**NNK**TTTCTTCAGGGCGAGG
7	FbFP_Q57Xmut	TCTATCAGGACGCACGTTTTCTT**NNK**GGCGAGGATCA
8	FbFP_R70Xmut	GGGCATCGCAATTATC**NNK**GAGGCGATCCG
9	FbFP_N85Xmut	GCCAGGTGCTGCGC**NNK**TACCGCAAAGACG
10	FbFP_W94Xmut	AGACGGCAGCCTGTTC**NNK**AACGAGTTGTCCATC
11	FbFP_Y112Xmut	GACCAGCTGACCTAC**NNK**ATCGGCATCCAGCG
12	FbFP_SeqPrimer	GCATCACCATCACCATCACG

*BamHI *and *HindIII *restriction sites in the forward and reverse primers used for amplification of the FbFP gene are underlined. The NNK degenerate oligonucleotide in the mutagenic primers is depicted in bold font. The sequencing primer (FbFP_SeqPrimer) is specific to the pQE80L vector and anneals immediately downstream of the RGS epitope.

### Site saturation mutagenesis

Saturation mutagenesis at the nine amino acid targets was performed as described in Materials and Methods. For each target mutant, we screened up to 180 isolated colonies using fluorescence spectrophotometry, which corresponds to approximately sixfold coverage of the mutation space (4 × 4 × 2 = 32 possible mutations with NNK degenerate codon) and was deemed to be a statistically significant sample size by the CASTER program [49]. CASTER is an open source worksheet that enables estimation of the number of colonies that are required to be screened in order to ensure a 95% coverage of all possible variants resulting from site saturation mutagenesis [49]. FbFPs are significantly less bright compared to most proteins in the GFP-family (~13% and ~4% as bright as GFP and the spectrally equivalent mTFP1, respectively [50]). Therefore, we determined that at least a twofold enhancement in brightness or a substantial spectral shift would be useful criteria for obtaining fluorescent reporters of practical relevance. In this way, we defined beneficial mutants as cells displaying at least a twofold enhancement in fluorescence emission and/or a 10 nm or longer shift in emission wavelength. Mutants that scored in the 96-well plate assays and shake flask-based screens were isolated and purified, and the fluorescence spectra of the purified mutant proteins were compared against purified wild type FbFP. Direct comparison of fluorescence spectra between purified protein preparations ensures that improvements in spectral properties are the direct result of mutations in the open reading frame and do not arise due to improved cellular production of the mutant proteins (*e.g.*, enhanced emission due to improved protein production by codon adaptation at a mutated site). Only mutants satisfying the improvement criteria in all three screens (96-well plate, shake flask, and purified protein) were classified as beneficial mutants that show significant improvement relative to the wild type protein.

Using this approach, we found that mutations at six of the nine target amino acids (D52, R54, Q57, R70, W94, and Y112) yielded predominantly neutral mutant proteins, with ~82% clones showing fluorescence emission spectra identical to the wild type protein. However, mutations at the other three amino acid targets (F37, A53, and N85) resulted in a smaller fraction (40%) of clones identical to the wild type protein, with the remaining 60% colonies showing greatly diminished fluorescent yields (Table 2). We hypothesize that neutral mutants represent amino acid substitutions that minimally perturb interactions between the protein and the FMN chromophore, whereas mutants showing severely diminished fluorescence represent amino acid substitutions that quench fluorescence by affecting the photophysical properties of FMN, by weakening the association between FMN and the protein, or by compromising efficient protein folding. Sequencing of randomly selected mutants confirmed that amino acid substitutions were faithfully incorporated in a random fashion at the target site without amino acid bias due to the use of degenerate codons.

**Table 2:** Classification of mutants

**Mutation**	**Beneficial**	**Deleterious**	**Neutral**
F37X	2	71	103
D52X	0	42	138
A53X	0	60	57
R54X	0	28	152
Q57X	0	30	150
R70X	0	18	120
N85X	0	141	20
W94X	0	15	58
Y112X	0	31	149

Mutations were introduced in the wild type FbFP by site saturation mutagenesis and mutants were classified as beneficial, neutral, or deleterious based on their fluorescent properties. Specifically, **beneficial mutants **exhibit at least a twofold enhancement in brightness of fluorescence emission (or a 10 nm or greater spectral shift), **deleterious **mutants have substantially diminished (greater than twofold) fluorescence emission compared to the wild type FbFP, and **neutral **mutants are similar to the wild type proteins with respect to brightness and emission wavelength.

### Isolation of enhanced brightness F37S and F37T mutants

**Figure 5:**
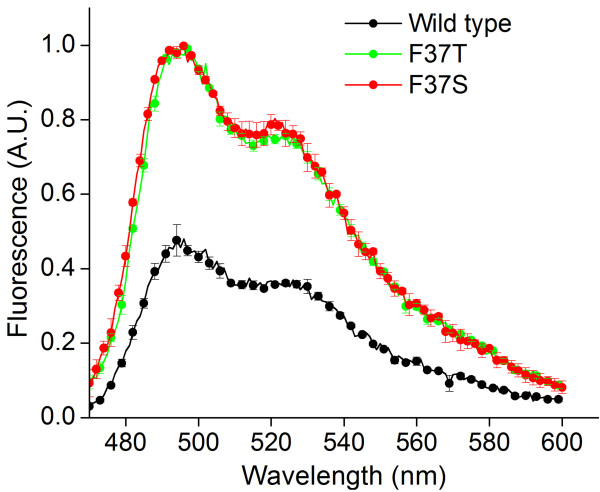
**Emission spectra of purified F37S and F37T mutant proteins. **FbFP F37S and F37T mutant proteins have approximately twofold enhanced emission yields at 500 nm, similar to fluorescence measurements in whole cell based assays. Excitation was performed at 450 nm, and values are normalized to the protein concentration as determined by the Bradford assay.

**Figure 6:**
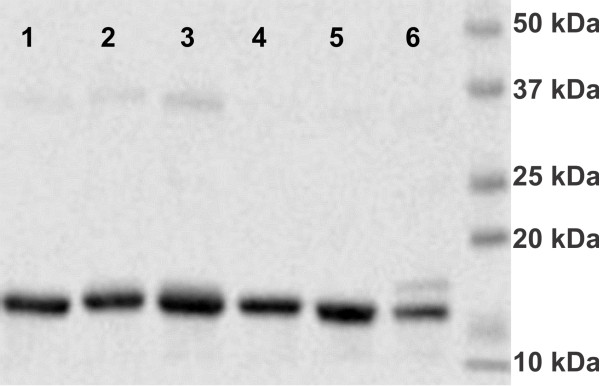
**SDS PAGE analysis for wild type and mutant FbFPs. **Lane 1: F37W, Lane 2: F37E, Lane 3: F37Y, Lane 4: F37T, Lane 5: F37S, Lane 6: Wild type FbFP. Selected mutants (F37W, F37E, F37Y, F37S, and F37T) of the FbFP F37X saturation mutagenesis library were purified and run on a 10% denaturing polyacrylamide gel (160 V, 45 minutes). Fluorescence enhanced mutants FbFP F37S and F37T comigrate with the wild type band (WT) at an approximate molecular mass of 17 kDa. Additional mutants correspond to neutral (F37E, lane 2) or deleterious mutants (F37W, lane 1 and F37Y, lane 3).

We isolated two mutants from the saturation mutagenesis library that showed spectral improvements compared to the wild type FbFP. In particular, we discovered two mutants of the same amino acid (phenylalanine residue at position 37 in wild type FbFP) that yield a twofold enhancement in peak fluorescence intensity (at 495 nm) compared to the wild type protein (Additional file 1, Figure 5). The excitation and emission spectral shapes of the mutant proteins were unchanged relative to the wild type protein. We verified that the bright mutants fluoresced in anaerobic conditions with emission at 495 nm greater than that of the wild type protein (Additional file 2). Mutant proteins are readily expressed in bacterial expression hosts and comigrate with the wild type FbFP protein in denaturing gels (Figure 6). Fluorescence measurements on purified mutant proteins revealed a similar twofold enhancement in peak fluorescence intensity (Figure 5), which agrees well with whole cell fluorescence assays. Moreover, we confirmed that the F37 amino acid was replaced by a serine and a threonine residue in the two beneficial mutants (F37S and F37T, respectively) using DNA sequencing. We further subjected the brighter of the two mutants FbFP F37S to random mutagenesis using error-prone PCR with a low mutation frequency (≈ 1–2 amino acid mutations per protein). Colonies from the random mutagenesis library were cloned in *E. coli* DH5α cells and plated on LB-ampicillin plates. We screened approximately 30,000 colonies using automated image analysis. However, we were unable to identify mutants with substantial improvements in brightness relative to the FbFP F37S parent protein (Additional file 3).

### Characterization of bright mutants F37S and F37T

In order to elucidate a biochemical and biophysical basis for the improved fluorescence observed in the F37S and F37T mutants, we characterized these beneficial mutants with respect to their oligomeric structure, quantum yield, and the fraction of FMN-bound holoprotein in solution. Fluorescence properties of the beneficial mutants F37S and F37T can be affected by: 1) changes in the oligomeric state of the mutants, 2) changes in the dissociation constant of the FMN chromophore, which can affect the levels of fluorescent holoprotein (FMN chromophore + FbFP apoprotein) for mutants relative to the wild type protein, and/or 3) altered photophysical interactions between the FMN chromophore and the amino acid at the mutation site.

**Figure 7:**
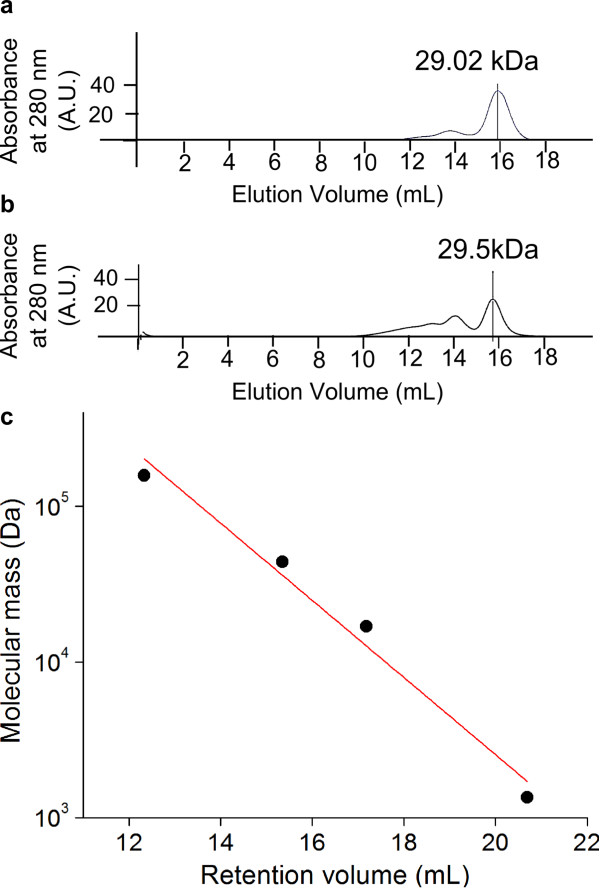
**Oligomeric state of improved FbFP F37S mutant. **Oligomeric states of native and mutant FbFPs were determined by size exclusion chromatography. Chromatograms of (**a**) wild type FbFP and (**b**) FbFP F37S from size exclusion chromatography. The F37S mutant and wild type FbFP were run through the Superdex 200 column under conditions identical to those used for the protein standards. FbFP F37S and wild type FbFP elute at similar volumes, thereby confirming that the native and mutant proteins have identical oligomeric conformations (dimeric). (**c**) Calibration plot for estimation of molecular mass using size exclusion chromatography. Globular proteins of known molecular weights were loaded onto a Superdex 200 gel filtration column and protein elution volumes corresponding to peak absorbance at 280 nm were recorded. A semi-logarithmic plot of molecular weight against elution volume yielded a straight line whose slope was used to calculate the molecular mass of the FbFP wild type and mutants.

We characterized the biochemical and photophysical properties of the beneficial mutants to determine the origins of the enhanced fluorescence properties, and the results are summarized in Table 3. First, we verified that the F37S and F37T mutations do not alter the oligomeric state of the protein. We found that wild type FbFP and the mutants exist predominantly as dimers in solution (Figure 7). Next, we estimated the fraction of fluorescent holoprotein in solution (*f*_*holo*_) based on absorption measurements at 450 nm using purified protein preparations (Materials and Methods). The bound FMN cofactor in holo FbFPs is responsible for absorption at 450 nm. Consequently, we do not expect the apo FbFP to exhibit significant absorption at 450 nm. Indeed, mutations in flavin-binding photosensory proteins that interfere with FMN binding have been shown to concomitantly eliminate activation of the photocycle by absorption of light at 450 nm [34]. In addition, we verified the absence of substantial 450 nm absorption using apo FbFP that had been deflavinated using urea denaturation and subsequently refolded to yield the apo protein. We calculated the fraction of fluorescent FMN-bound holoprotein for the wild type and mutant proteins from absorption measurements at 450 nm (which yields the concentrations of holoprotein) and from a Bradford assay (which yields the total protein concentration, including holoprotein and apoprotein forms). The fraction of holoprotein for the F37S and F37T mutants are *f*_*holo,F37S*_ = 0.49 ± 0.01 and *f*_*holo,F37T*_ = 0.45 ± 0.03, respectively, which represent 1.5 ± 0.04-fold and 1.4 ± 0.07-fold enhancements in fraction of holoprotein compared to wild type FbFP (*f*_*holo,WT*_ = 0.33 ± 0.01). Therefore, the improved association with FMN in the mutant proteins partially contributes to the observed twofold enhancement in emission intensity in the mutants.

**Table 3:** Characteristics of improved FbFP F37S and F37T mutants

**Property**	**FbFP**	**FbFP F37S**	**FbFP F37T**
Oligomeric state	1.81 (dimer)	1.78 (dimer)	1.87 (dimer)
Quantum yield	0.17 [21]	0.30 ± 0.01	0.24 ± 0.01
Fraction of holoprotein	0.33 ± 0.01	0.49 ± 0.01	0.45 ± 0.03

Enhanced brightness F37S and F37T mutants were characterized with respect to their oligomeric state, holoprotein fraction, and quantum yield in order to elucidate a biochemical and biophysical basis for the origin of improved fluorescence in these mutants. Specifically, we identified higher quantum yield and improved association with FMN in the mutant proteins as key contributors to their brighter fluorescence emission. Standard errors were calculated from at least four replicate measurements.

**Figure 8:**
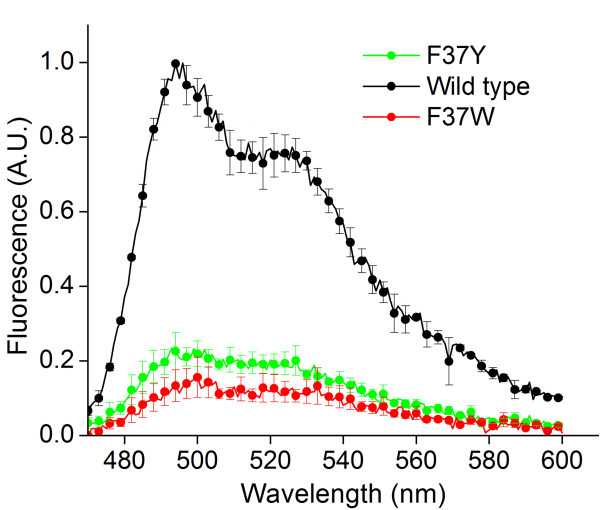
**Emission spectra of purified F37Y and F37W mutant proteins. **F37Y and F37W mutants show severely quenched fluorescence emission yields at 500 nm relative to the wild type protein. Samples were excited at 450 nm, and all values were normalized to protein concentrations as determined by the Bradford assay.

Finally, we experimentally measured the fluorescence quantum yields for the mutant proteins and the wild type protein (Materials and Methods). In these measurements, FMN was used as a standard with known quantum yield (*QY*_*FMN*_ = 0.27). We determined quantum yields for the F37S and F37T mutants to be *QY*_*F37S*_ = 0.30 ± 0.01and *QY*_*F37T*_ = 0.24 ± 0.01, which are 1.8 ± 0.03 and 1.4 ± 0.04-fold larger than the wild type protein (*QY*_*WT*_ = 0.17 [39]). Quantum yield represents the efficiency with which a single molecule of the fluorescent holoprotein converts excitation photons into fluorescence emission, which is independent of the fraction of fluorescent holoprotein in solution. Therefore, the increase in the quantum yields of the beneficial mutants F37S and F37T cannot be explained by improved association between FbFPs and FMN, because the nonfluorescent apoprotein fraction does not contribute to the emission spectrum or absorption at 450 nm. However, quantum yield can be affected by the nature of photophysical interactions between the FMN chromophore and nearby amino acids in FbFPs. It is noteworthy that in both enhanced fluorescence mutant proteins, the mutated residue is an aromatic amino acid in close proximity to the FMN chromophore. Aromatic amino acids are capable of a wide range of interactions with buried ligands, including excited state electron transfer in the protein nanospace [51] and π-π stacking interactions with the isoalloxazine ring of FMN, which can quench fluorescence emission. Indeed, the flavin analog flavin adenine dinucleotide (FAD) shows severely quenched fluorescence due to intramolecular π stacking interactions [52]. To further probe for the possibility of π-π stacking interactions in FbFPs, we generated mutants with other aromatic amino acids at the F37 site. We observed that substitution of phenylalanine at this position with tyrosine (F37Y) or tryptophan (F37W) results in a heavily quenched fluorescence (Figure 8), thereby suggesting that aromatic amino acids at position 37 in FbFP are likely to be involved in fluorescence quenching photophysical interactions with the isoalloxazine ring. Based on these results, we hypothesize that substitution of the phenylalanine residue with polar amino acids at a chromophore proximal position in the wild type FbFP lessens the degree of fluorescence quenching by preventing stacking interactions between the FMN chromophore and the amino acid, thereby resulting in higher quantum yields in the mutants. Moreover, substitution of phenylalanine by similar polar amino acids (serine and threonine) in the two mutants may reflect enhanced structural stabilization of the mutant protein mediated by interactions between the neighboring amino acids and the hydroxyl group of serine or threonine. Based on these observations, we conjecture that enhanced fluorescence emission in the F37S and F37T mutants results from two contributing factors: 1) stronger binding between the FMN fluorophore and FbFP in the mutants, thereby resulting in a higher fraction of FMN-bound fluorescent protein in solution, and 2) reducing the degree of fluorescence quenching interactions between the aromatic amino acid phenylalanine and FMN in the mutants, thereby resulting in increased quantum yields.

## Conclusions

In this work, we successfully employed site saturation mutagenesis to isolate two fluorescence enhanced mutants of an anaerobic fluorescent protein from *Pseudomonas putida*. Although directed evolution of a plant-based flavin binding fluorescent phototropin for enhanced fluorescence in plant cells has been described previously [22], our study utilizes laboratory evolution to develop flavin-based anaerobic fluorescent proteins for prokaryotic expression. We identified FMN-proximal aromatic amino acids as particularly important in affecting the spectral response of FbFPs by directly affecting the quantum yield of the bound FMN cofactor as well as the strength of FMN binding. Future efforts aimed at improving the brightness of FbFPs can be directed at enhancing both the quantum yield as well as the holoprotein fraction by engineering stronger binding mutants. Although we were unable to further enhance the brightness of the F37S mutant using error-prone PCR (Additional file 3), we speculate that additional improvements in FbFP fluorescence may be achieved by employing alternative evolution strategies such as family shuffling. In future work, we are investigating these options to generate improved variants of the F37S mutant. Furthermore, aromatic amino acids in other FbFPs (*B. subtilis, A. thailana*) could be considered as mutagenesis “hot spots” to engineer and fine-tune FbFP fluorescence. We anticipate that further engineering of anaerobic fluorescent proteins for spectral enhancements will enable new studies of real time gene expression, protein localization, and dynamic protein interactions in anaerobic bioprocesses with broad implications in systems and synthetic biology, biotechnology, and biological engineering. In this way, we anticipate that our study will furnish an experimental and theoretical basis to enable further engineering of FbFPs, which are currently at an early stage of development.

## Methods

### Bacterial strains and growth media

*E. coli* BLR (DE3) expression strains (EMD Chemicals) were used for protein expression. *E. coli* DH5α cells were used for cloning and propagation of the wild type flavin-binding fluorescent protein (FbFP) gene from *Pseudomonas putida*. Cells were grown with vigorous shaking (200 r.p.m.) at 37°C in Lennox broth (10 g/L tryptone, 5 g/L yeast extract, 5 g/L NaCl) supplemented with ampicillin at 100 μg/mL. For growth in solid phase, cells were grown on solid 1.5% agar plates containing LB medium, and plates were incubated at 37°C for 20–48 hours. Growth in high-brim 96-well plates (Axygen) was achieved using 800 μL cell culture volumes. The plates were sealed with oxygen permeable sealing mats (Axygen) to minimize evaporation and contamination. Antibiotics and salts were purchased from Sigma-Aldrich (St. Louis, MO) and were of the highest possible purity. Tryptone, agar, and yeast extract were purchased from Fisher Scientific (Pittsburgh, PA).

### Cloning of FbFP gene from *Pseudomonas putida*

The *Pseudomonas putida* KT2440 gene coding for the FbFP [RefSeq ID: NP_744883.1] was synthesized by GenScript (Piscataway, NJ). The synthetic gene was cloned into the pQE80L expression vector (Qiagen) using *BamHI* and *HindIII* restriction enzymes, following standard protocols [53], and the construct was designated pQE80L-FbFP. DNA sequences for all primers used in this work are listed in Table 1. The pQE80L vector appends a hexahistidine tag to the N-terminus of the protein, thereby facilitating protein purification on nickel chelating columns. The expression constructs were transformed to *E. coli* DH5α cells by heat shock transformation at 42°C, and transformants were selected on LB plates solidified with agar at 1.5% and supplemented with ampicillin. Plasmids were isolated from *E. coli* DH5α transformants (Qiagen Miniprep kit) and were used to transform *E. coli* BLR (DE3) cells for protein expression. Restriction enzymes were purchased from New England Biolabs (Ipswich, MA), and primers were synthesized by Integrated DNA Technologies (Coralville, IA).

### Directed evolution of FbFP from *Pseudomonas putida*

A schematic of the protocol for directed evolution is presented in Additional file 4. Mutations were engineered in wild type FbFP using site-saturation mutagenesis, followed by transformation into *E. coli* BLR (DE3) expression strains. Expression vectors and induction conditions were optimized for high transformation efficiencies and enhanced expression levels of the desired proteins. Specifically, we cloned the wild type FbFP gene in three different expression vectors: pQE80L (Qiagen), pET28a(+) (Novagen), and pET*28a(+), where the latter is an expression vector constructed in-house by mutating the promoter for the lac repressor in pET28a(+) to induce high levels of expression of the repressor protein [54]. *E. coli* BLR cells were transformed with the aforementioned expression constructs, and the transformation efficiencies were assessed. Transformed cells were induced for FbFP expression using three different strategies: IPTG induction for 8 hours, 16 hours, and autoinduction [55]. For these three plasmid constructs, fluorescence levels were measured and compared between induced and uninduced cells using these three different induction strategies. We observed that the pQE80L construct consistently yielded the highest transformation efficiencies as well as the largest normalized fluorescence levels in induced cells (Additional file 5). Therefore, the pQE80L vector was used to clone the mutant libraries. Screening was implemented in a 96-well format in liquid media and was performed approximately 8 to 12 hours after induction of protein expression with IPTG. Subsequent to the initial screen, improved mutants were carried forward and further assayed by fluorescence measurements in shake flask cultures and using purified protein preparations.

### Site saturation mutagenesis

Site saturation mutagenesis was accomplished with the QuikChange^TM^ Multi Site Directed Mutagenesis kit (Agilent Technologies) using degenerate oligonucelotides that substitutes the targeted codon with an NNK triplet (N: any nucleotide, K: G or T). Briefly, 300 ng of the pQE80L-FbFP expression vector was used as a template in a 50 μL ligation-during-amplification PCR [56] comprising an initial denaturation at 95°C for 1 minute and 30 cycles of 95°C for 1 minute, 55°C for 1 minute, 65°C for 12 minutes and a final extension at 65°C for 15 minutes. The products of the reaction are single stranded circular plasmids harboring a degenerate NNK substitution at the desired codon. The reaction was digested with *DpnI* as before to eliminate plasmids bearing the native FbFP gene. Plasmids were transformed to *E. coli* BLR (DE3) cells by heat shock transformation at 42°C. Transformants were selected by ampicillin resistance. Primer sequences used to generate mutations for site saturation mutagenesis are listed in Table 1.

### Screening mutant libraries for improved variants

Transformants from the site saturation mutagenesis library were plated on LB-ampicillin plates without IPTG. Following incubation for 20 hours at 37°C, single colonies were picked from the plates with sterile toothpicks and inoculated in 800 μL LB-ampicillin media in high-brim 96-well plates and grown for 16 hours. Cells from the overnight cultures were diluted 100-fold in fresh LB media and grown for an additional 2 hours before inducing with 1 mM IPTG for 8–12 hours. Fluorescence measurements were then conducted in optically clear round bottom 96-well plates (BrandTech Scientific, Essex, CT) in a spectrofluorometer (Cary Eclipse Fluorescence Spectrophotometer, Varian). Fluorescence emission scans spanning 470 nm to 600 nm wavelength range (1 nm resolution) were recorded from each well at two excitation wavelengths (450 nm and 500 nm). Background fluorescence was measured in uninduced cells bearing wild type pQE80L-FbFP and was subtracted from all readings. The resulting spectra were smoothed by 3^rd^ order Savitzky Golay filtering with a frame size of 5. Selected mutants were subject to further spectrofluorometric analyses using shake flask cultures and purified protein preparations. Induction of protein expression and subsequent fluorescence measurements were conducted as previously described. Analyses of fluorescence spectra were performed using custom codes written in MATLAB version 7.10 (MathWorks) and are available on request.

### Homology Modeling of the FbFP from *Pseudomonas putida*

A predetermined structure of the *Bacillus subtilis* blue light photoreceptor, YTVA [PDB: 2PR5_A] was used as a template to model the structure of the *Pseudomonas putida* FbFP. Homology modeling was implemented in the Swiss PDB Viewer version 4.0 [57]. An initial raw fit was first constructed by the iterative magic fit option. Amino acids showing steric clashes with the peptide backbone were corrected by iterative simulated annealing. The structure was energy minimized using the Gromos96 force field, and the final structure was validated by measuring the root mean-squared deviation of the Cα backbone and by inspecting the Ramachandran plot. Amino acids (except glycine and proline) lying in the prohibited areas of the Ramachandran plot (specifically, Y50, D107, H13, A21, I48, V119) were omitted from further consideration. As further validation, we compared amino acids located in a chromophore-proximal 0.4 nm cavity surrounding FMN in the modeled structure with chromophore-proximal amino acids in the known crystal structures of homologous LOV domain proteins from *Chlamydomonas reinhardtii* [PDB: 1N9L_A] and *Arabidopsis thaliana* [PDB: 2Z6D_A]. In all cases, an 80-85% agreement was obtained between the identities of the amino acids in the 0.4 nm cavity surrounding FMN in the modeled and the known X-ray diffraction structures (Additional file 6).

### Protein expression and purification

An isolated *E. coli* BLR colony expressing the pQE80L-FbFP construct was inoculated in 5 mL LB-ampicillin medium and grown for 16 hours. Cells from the overnight culture were diluted 100-fold in 500 mL fresh LB-ampicillin medium in a 2 L shake flask and induced with IPTG at 1 mM concentration in the mid-exponential phase (A_600_ ≈ 0.5) of cell growth. Protein expression was continued for 5 hours at 37°C. Protein purification was achieved by immobilized nickel-affinity chromatography (Qiagen Ni-NTA resin) and anion-exchange chromatography (HiTrap Q Sepharose, GE Healthcare). Briefly, following IPTG induction for 5 hours, *E. coli* cells were centrifuged at 5000g for 15 minutes and resuspended in Buffer A (25 mM sodium phosphate, 500 mM sodium chloride, pH 7.4). The cells were incubated with lysozyme at 1 mg/mL at 4°C for 30 minutes and lysed with an ultrasonicator. Cell debris was removed by centrifugation at 10,000g for 30 minutes, and the supernatant was supplemented with imidazole at 10 mM and incubated with 4 mL nickel-chelating resin at 4°C for 1 hour. Non-specifically bound proteins were removed by washing the column with 50 mL Buffer B (25 mM sodium phosphate, 500 mM sodium chloride, 40 mM imidazole, pH 7.4) and the protein was eluted with 25 mL Buffer C (25 mM sodium phosphate, 500 mM sodium chloride, 500 mM imidazole, pH 7.4). Protein-containing fractions were visibly fluorescent and were pooled and diluted to reduce NaCl concentration to 200 mM and loaded onto a 5 mL HiTrap Q Sepharose anion exchange column (GE Healthcare). At this NaCl concentration, the protein was strongly bound the anion-exchange column. The bound protein was washed with 25 mL buffer D (25 mM sodium phosphate, 200 mM sodium chloride, pH 7.4), and eluted in 25 mL Buffer E (25 mM sodium phosphate, 1 M sodium chloride, pH 7.4. Protein fractions were assayed by denaturing polyacrylamide gel electrophoresis (SDS PAGE) (Additional file 7) and spectrofluorometry. Purified proteins were stored at 4°C and were stable for at least a week under these conditions. IPTG was purchased from Sigma-Aldrich (St. Louis, MO). Mutant FbFP proteins were purified exactly as described for the wild type FbFP.

### Determination of oligomeric state of proteins

Oligomeric states of the wild type and mutant FbFP proteins were assessed using size exclusion chromatography using a Superdex 200 column in an AKTA FPLC system (GE Healthcare). The column was calibrated with globular protein standards of known molecular mass, including bovine thyroglobulin (670 kDa), bovine γ-globulin (158 kDa), chicken ovalbumin (44 kDa), and horse myoglobin (17 kDa) (Figure 7). Purified FbFP, F37S, and F37T mutant proteins were loaded onto a Superdex 200 column in 20 mM Tris, 1M NaCl buffer at a pH of 8.0, and elution volumes corresponding to the peaks in the 280 nm absorption chromatogram were recorded. Net molecular mass was then estimated and oligomeric state determined by dividing the net mass by the calculated mass of a monomer (16.3 kDa).

### Determination of quantum yield

For quantum yield measurements, varying concentrations of the proteins (in the range 0.1-1 mg/mL) were excited with 450 nm light in a spectrofluorometer (Cary Varian) and the emission spectrum was recorded between 470 and 600 nm. The emission spectrum was then integrated and normalized by the absorbance at 450 nm. In an analogous fashion, we recorded the emission spectrum and absorption at 450 nm of FMN in the concentration range 6.2-25 μM, which was used as a standard with known quantum yield to calculate FbFP quantum yields by the equation:

(1) $$QY_{\text{FbFP}=}\frac{\int_{470nm}^{600nm}\left(F_{\text{FbFP}}\right)d\lambda }{A_{\text{FbFP}}}.\frac{A_{\text{FMN}}}{\int_{470nm}^{600nm}\left(F_{\text{FMN}}\right)d\lambda }.QY_{\text{FMN}}$$

where *QY* is the fluorescence quantum yield, *F* is the fluorescence emission intensity, and *A* is the absorbance. Subscripts *FMN* and *FbFP* refer to the FMN standard and the FbFP samples of unknown quantum yields, respectively. Quantum yields were estimated using highly purified proteins, which contain negligible free FMN. Furthermore, we observed that the quantum yield in wild type FbFPs from *P. putida* and *B. subtilis* cannot be increased by addition of FMN to solutions of purified proteins (data not shown). Therefore, the concentration of free FMN in solutions of purified wild type and mutant proteins is assumed to be negligible.

### Calculation of holoprotein fraction

FbFPs are fluorescent only in their FMN-bound or holoprotein form. Absorbance at 450 nm is determined exclusively by the holoprotein and unaffected by the apoprotein fraction. Therefore, concentration of holoprotein was calculated from the Beer-Lambert equation:

(2) $$C_{\text{holo}}=\frac{A_{450\;nm}}{\varepsilon \;l}\;$$

where *C*_*holo*_ is the concentration of the holoprotein, ε is the molar absorption coefficient of FMN (12500 M^-1^cm^-1^), and *l* is the cuvette path length. The holoprotein concentration was further divided by the total protein concentration (measured by the Bradford assay) to elucidate the fraction of fluorescent holoprotein in solution. Holoprotein fractions were calculated using highly purified proteins, which contain negligible FMN in solution. Moreover, we observed that the fluorescent holoprotein fraction in wild type FbFPs from *P. putida* and *B. subtilis* cannot be increased by addition of FMN to solutions of purified protein (data not shown). Therefore, the concentration of free FMN in solutions of purified wild type and mutant proteins is assumed to be negligible.

### Gene sequencing

All sequencing reactions were performed at the Core DNA Sequencing Facility at the Roy J. Carver Biotechnology Center, University of Illinois at Urbana-Champaign.

## Abbreviations

FbFP: Flavin-binding fluorescent protein; FMN: Flavin mononucleotide; GFP: Green fluorescent protein; LOV: Light oxygen voltage; IPTG: Isopropyl-β-D-thiogalactopyranoside; SDS-PAGE: Sodium dodecyl sulfate polyacrylamide gel electrophoresis; UV: Ultraviolet.

## Competing interests

The authors declare that they do not have any competing interests.

## Authors' contributions

CMS and AM designed experiments and analyzed data. AM, KBW, and JW performed the experiments. CMS and AM wrote the paper. All authors read and approved the final manuscript.

## Supplementary Material

Additional file 1 **Emission spectra of *E. coli *cells expressing improved mutants. ***E. coli *cells expressing FbFP F37S and F37T mutants have approximately twofold enhanced peak emission yields, relative to cells expressing the wild type protein. Excitation was performed at 450 nm and emission spectra were scanned between 470 and 600 nm. Whole cell emission spectra tend to be noisy owing to the effects of cellular autofluorescence and the inherent dimness of the fluorescent proteins. Click here for file

Additional file 2 **Emission spectra of anaerobically cultured *E. coli *cells expressing improved mutants. ***E. coli *cells expressing FbFP F37S and F37T mutants and cultivated under anaerobic conditions have enhanced peak emission yields, relative to cells expressing the wild type protein in anaerobic conditions. Excitation was performed at 450 nm and emission spectra were scanned between 470 and 600 nm. For anaerobic cultivation, *E. coli* cells were grown in M9 medium supplemented with glucose (20 mM) as the carbon source and nitrate (20 mM) as an electron acceptor. Anaerobic conditions were established by growing the cells in sealed air-tight Balch tubes, which were completely filled with the growth medium and further degassed by applying vacuum for 30 minutes. As protein expression is substantially reduced in anaerobic conditions, the whole cell cultures were concentrated approximately 18-fold prior to spectral analysis. Click here for file

Additional file 3 **Error-prone PCR mutagenesis of FbFP F37S bright mutant. **Herein, we describe the application of random mutagenesis by error-prone PCR in an effort to improve fluorescence emission of the F37S bright mutant. Briefly, we screened 27,425 colonies from the error-prone PCR library but were unable to isolate mutants with significant improvement in brightness. Click here for file

Additional file 4 **Schematic of directed evolution pipeline. **Genes encoding for FbFP are cloned into an IPTG-inducible pQE80L expression vector, followed by site saturation mutagenesis to introduce mutations at specific sites. Mutants are then grown overnight on LB-agar plates and subsequently screened in a 96-well format using spectrofluorometry. Beneficial mutants are identified, selected, and carried forward for growth in shake flask cultures, followed by analysis by fluorescence. Finally, a subset of beneficial mutants are isolated, purified and further analyzed by spectrofluorometry using purified protein preparations. Click here for file

Additional file 5 **Optimizing conditions for laboratory evolution of FbFP. **(a) Expression levels of wild type FbFP protein cloned in *E. coli *cells and expressed from a T5 or a T7 promoter (pQE80L and pET28a(+) vectors respectively) and using three different induction strategies: IPTG induction for 8 hours, 16 hours, and autoinduction. (b) Transformation efficiencies for pQE80L, pET28a(+) and pET*28a(+) expression vectors. pET*28a(+) is a variant of pET28a(+) engineered to express higher levels of the lac repressor, thereby minimizing leaky expression. The pQE80L expression system under IPTG induction outperformed the others in exhibiting higher transformation efficiency and stronger levels of expression. Click here for file

Additional file 6 **Sequence alignment between FbFP and LOV domains from *Chlamydomonas reinhardtii * and *Arabidopsis thaliana *phototropins. **Selection of organisms for sequence alignment was motivated by the availability of high quality structures for these LOV proteins. For each alignment, we compared the extent of agreement between amino acids in the chromophore proximal cavity (up to 0.4 nm from FMN) in the homology modeled structure of FbFP and the known crystal structures of other proteins. The chromophore proximal amino acids for each protein are shown in red. Similar amino acids occupying the FMN proximal position in the modeled FbFP structure and the crystal structures of *Chlamydomonas *and *Arabidopsis *phototropins (CrPhot and AtPhot respectively) are shown in green. The alignment shows close agreement between the amino acids in the chromophore proximal cavities of our homology model and the existing X-ray diffraction structures of LOV domain proteins from other organisms. Click here for file

Additional file 7 **SDS PAGE analysis of purified FbFPs . Purified FbFP fractions migrate as a ~17 kDa band on a 10% polyacrylamide gel. **The left-most lane corresponds to the molecular weight ladder. Lanes 1–5 correspond to protein fractions eluted from a two-step chromatographic separation comprising nickel-affinity chromatography and anion-exchange chromatography. Click here for file
