# Supplementary material for: Directed evolution of bright mutants of an oxygen-independent flavin-binding fluorescent protein from Pseudomonas putida
Source: J Biol Eng. 2012 Oct 24;6:20. doi: 10.1186/1754-1611-6-20 (PMC3488000; doi:10.1186/1754-1611-6-20)
Supplement: Additional file 1 — Emission spectra of E. coli cells expressing improved mutants. E. coli cells expressing FbFP F37S and F37T mutants have approximately twofold enhanced peak emission yields, relative to cells expressing the wild type protein. Excitation was performed at 450 nm and emission spectra were scanned between 470 and 600 nm. Whole cell emission spectra tend to be noisy owing to the effects of cellular autofluorescence and the inherent dimness of the fluorescent proteins. [file 1754-1611-6-20-S1.docx]

**Emission spectra of *E. coli* cells expressing improved mutants**


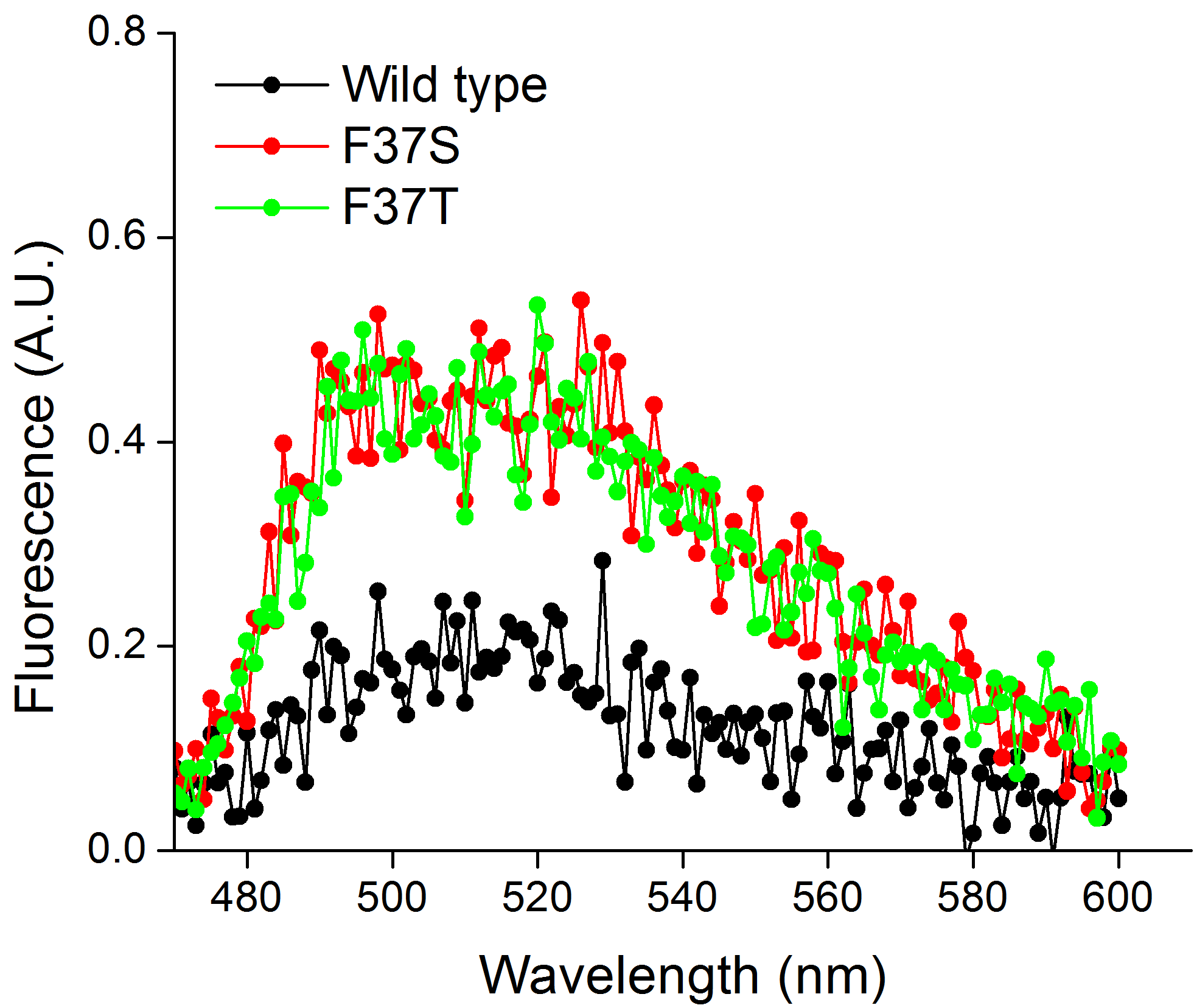


*E. coli* cells expressing FbFP F37S and F37T mutants have approximately twofold enhanced peak emission yields, relative to cells expressing the wild type protein. Excitation was performed at 450 nm and emission spectra were scanned between 470 and 600 nm. Whole cell emission spectra tend to be noisy owing to the effects of cellular autofluorescence and the inherent dimness of the fluorescent proteins.
