# Supplementary material for: Directed evolution of bright mutants of an oxygen-independent flavin-binding fluorescent protein from Pseudomonas putida
Source: J Biol Eng. 2012 Oct 24;6:20. doi: 10.1186/1754-1611-6-20 (PMC3488000; doi:10.1186/1754-1611-6-20)
Supplement: Additional file 2 — Emission spectra of anaerobically cultured E. coli cells expressing improved mutants. E. coli cells expressing FbFP F37S and F37T mutants and cultivated under anaerobic conditions have enhanced peak emission yields, relative to cells expressing the wild type protein in anaerobic conditions. Excitation was performed at 450 nm and emission spectra were scanned between 470 and 600 nm. For anaerobic cultivation, E. coli cells were grown in M9 medium supplemented with glucose (20 mM) as the carbon source and nitrate (20 mM) as an electron acceptor. Anaerobic conditions were established by growing the cells in sealed air-tight Balch tubes, which were completely filled with the growth medium and further degassed by applying vacuum for 30 minutes. As protein expression is substantially reduced in anaerobic conditions, the whole cell cultures were concentrated approximately 18-fold prior to spectral analysis. [file 1754-1611-6-20-S2.docx]

**Emission spectra of anaerobically cultured *E. coli* cells expressing improved mutants**


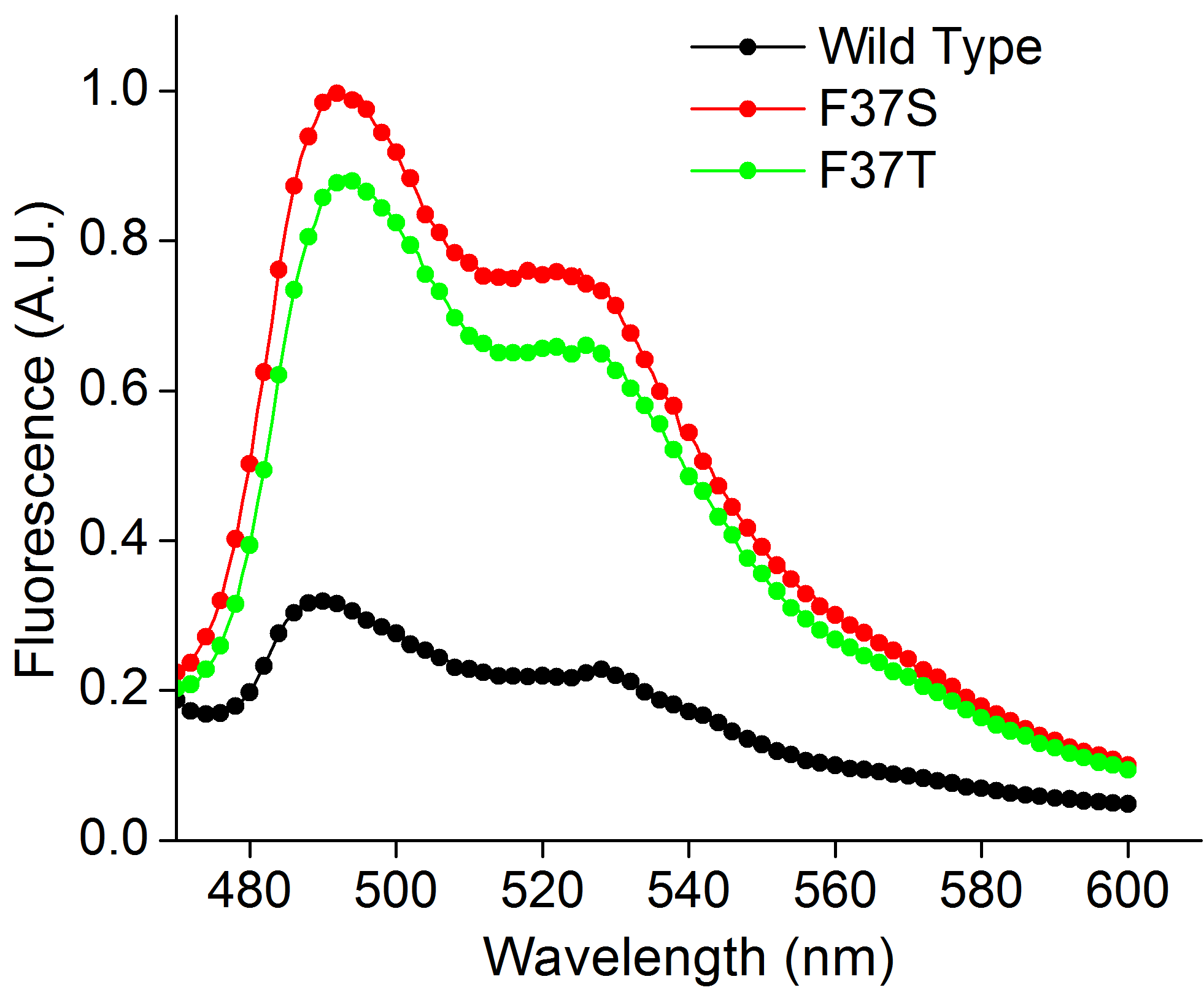


*E. coli* cells expressing FbFP F37S and F37T mutants and cultivated under anaerobic conditions have enhanced peak emission yields, relative to cells expressing the wild type protein in anaerobic conditions. Excitation was performed at 450 nm and emission spectra were scanned between 470 and 600 nm. For anaerobic cultivation, *E. coli* cells were grown in M9 medium supplemented with glucose (20 mM) as the carbon source and nitrate (20 mM) as an electron acceptor. Anaerobic conditions were established by growing the cells in sealed air-tight Balch tubes, which were completely filled with the growth medium and further degassed by applying vacuum for 30 minutes. As protein expression is substantially reduced in anaerobic conditions, the whole cell cultures were concentrated approximately 18-fold prior to spectral analysis.
