# Supplementary material for: Directed evolution of bright mutants of an oxygen-independent flavin-binding fluorescent protein from Pseudomonas putida
Source: J Biol Eng. 2012 Oct 24;6:20. doi: 10.1186/1754-1611-6-20 (PMC3488000; doi:10.1186/1754-1611-6-20)
Supplement: Additional file 3 — Error-prone PCR mutagenesis of FbFP F37S bright mutant. Herein, we describe the application of random mutagenesis by error-prone PCR in an effort to improve fluorescence emission of the F37S bright mutant. Briefly, we screened 27,425 colonies from the error-prone PCR library but were unable to isolate mutants with significant improvement in brightness. [file 1754-1611-6-20-S3.docx]

**Error-prone PCR mutagenesis of FbFP F37S bright mutant**

**Materials and Methods**

**Error-prone PCR mutagenesis**

Error-prone PCR was performed with the Genmorph™ II EZClone Domain Mutagenesis Kit (Agilent Technologies) using primers specific for the FbFP gene, FbFP_ampl_fwd and FbFP_ampl_rev (**Table 1**). Briefly, the FbFP F37S mutant gene was amplified by PCR consisting of an initial denaturation step of 95 ^o^C for 2 minutes and 20 cycles of 95 ^o^C for 30 seconds, 64 ^o^C for 30 seconds and 72 ^o^C for 1 minute, followed by a final extension step at 72 ^o^C for 10 minutes. The template plasmid was eliminated by digestion with *DpnI* (New England Biolabs) at 37 ^o^C for 2 hours. The amplicon was purified (Qiagen PCR Cleanup Kit) and used as a megaprimer in a second high-fidelity ligation-during-amplification PCR with the pQE80L-FbFP expression vector as the template [1]. The denaturation step was performed at 95 ^o^C for 1 minute followed by 25 cycles of 95 ^o^C for 50 seconds, 60 ^o^C for 50 seconds, and 68 ^o^C for 14 minutes and a final extension at 68 ^o^C for 15 minutes. The products of the second PCR reaction are plasmids harboring randomly mutated variants of the FbFP F37S gene. These were further digested with *DpnI* for 3 hours at 37^o^C to eliminate methylated and hemimethylated plasmids bearing wild type FbFP. The mutant library was cloned in *E. coli* DH5α cells by electroporation.

**Screening of error-prone PCR mutants**

Transformants from the error-prone PCR library were plated on LB-agar plates supplemented with ampicillin at 100 µg/mL. The plates were incubated at 37 ^o^C for 20 hours. Colonies were then imaged using a UV illuminator. Images were analyzed using Cell Profiler and ImageJ to identify the brightest colonies. In this way, we screened a total of 27,425 colonies and identified 38 bright colonies. These colonies were further inoculated in Lennox broth in 96-well plates along with *E. coli* DH5α cells expressing the F37S mutant. Following overnight growth, the cells were rediluted in fresh medium (1% v/v dilution) supplemented with IPTG at 1 mM to induce protein expression. The induced cultures were harvested approximately 6 h. after inoculation (corresponding to an absorbance of ≈ 0.7 at 600 nm) and fluorescence emission was measured at 495 nm using a spectrofluorometer (Tecan M200). Whole cell fluorescence emission was normalized by the optical density at 600 nm and compared with the same values for cells expressing FbFP F37S.

**Results and Discussion**

Random mutagenesis by error-prone PCR is widely employed to generate mutant libraries of enzymes and fluorescent proteins for directed evolution [2-4]. In order to evaluate the potential of further improving FbFP fluorescence using this strategy, we subjected the brighter of the two discovered mutants, FbFP F37S to random mutagenesis using low-fidelity PCR. We selected reaction conditions to correspond to a low mutation frequency (≈ 1-2 amino acid mutations per protein), as recommended by the vendor (Agilent, Genemorph EZClone Domain Mutagenesis Technical Manual). Colonies were screened by automated image analysis in order to identify the brightest 38 colonies from a total of approximately 30,000 colonies. However, we were unable to isolate colonies with substantial improvements in brightness of emission compared to the FbFP F37S expressing cells. Nonetheless, we note that further improvements in spectral emission of FbFP can be potentially sought by tuning the mutation frequencies of random mutagenesis or through the application of alternative evolution strategies such as DNA shuffling. In future work, we are investigating these options to generate improved variants of the F37S mutant.

**References:**

1. Sawano A and Miyawaki A. **Directed evolution of green fluorescent protein by a new versatile PCR strategy for site-directed and semi-random mutagenesis**. Nucleic Acids Res 2000, **28**(16).
2. Crameri A, Whitehorn EA, Tate E, Stemmer WPC. **Improved green fluorescent protein by molecular evolution using DNA shuffling**. Nat Biotechnol 1996, **14**:315-319.
3. Biles BD, Connolly BA. **Low-fidelity Pyrococcus furiosus DNA polymerase mutants useful in error-prone PCR**. Nucleic Acids Res 2004, **32**(22)
4. Fradkov AF, Chen Y, Ding L, Barsova EV, Matz MV, Lukyanov SA. **Novel fluorescent protein from Discosoma coral and its mutants possesses a unique far-red fluorescence**. FEBS Lett 2000, **479**:127-130
