# Supplementary material for: Directed evolution of bright mutants of an oxygen-independent flavin-binding fluorescent protein from Pseudomonas putida
Source: J Biol Eng. 2012 Oct 24;6:20. doi: 10.1186/1754-1611-6-20 (PMC3488000; doi:10.1186/1754-1611-6-20)
Supplement: Additional file 4 — Schematic of directed evolution pipeline. Genes encoding for FbFP are cloned into an IPTG-inducible pQE80L expression vector, followed by site saturation mutagenesis to introduce mutations at specific sites. Mutants are then grown overnight on LB-agar plates and subsequently screened in a 96-well format using spectrofluorometry. Beneficial mutants are identified, selected, and carried forward for growth in shake flask cultures, followed by analysis by fluorescence. Finally, a subset of beneficial mutants are isolated, purified and further analyzed by spectrofluorometry using purified protein preparations. [file 1754-1611-6-20-S4.docx]

**Schematic of directed evolution pipeline**


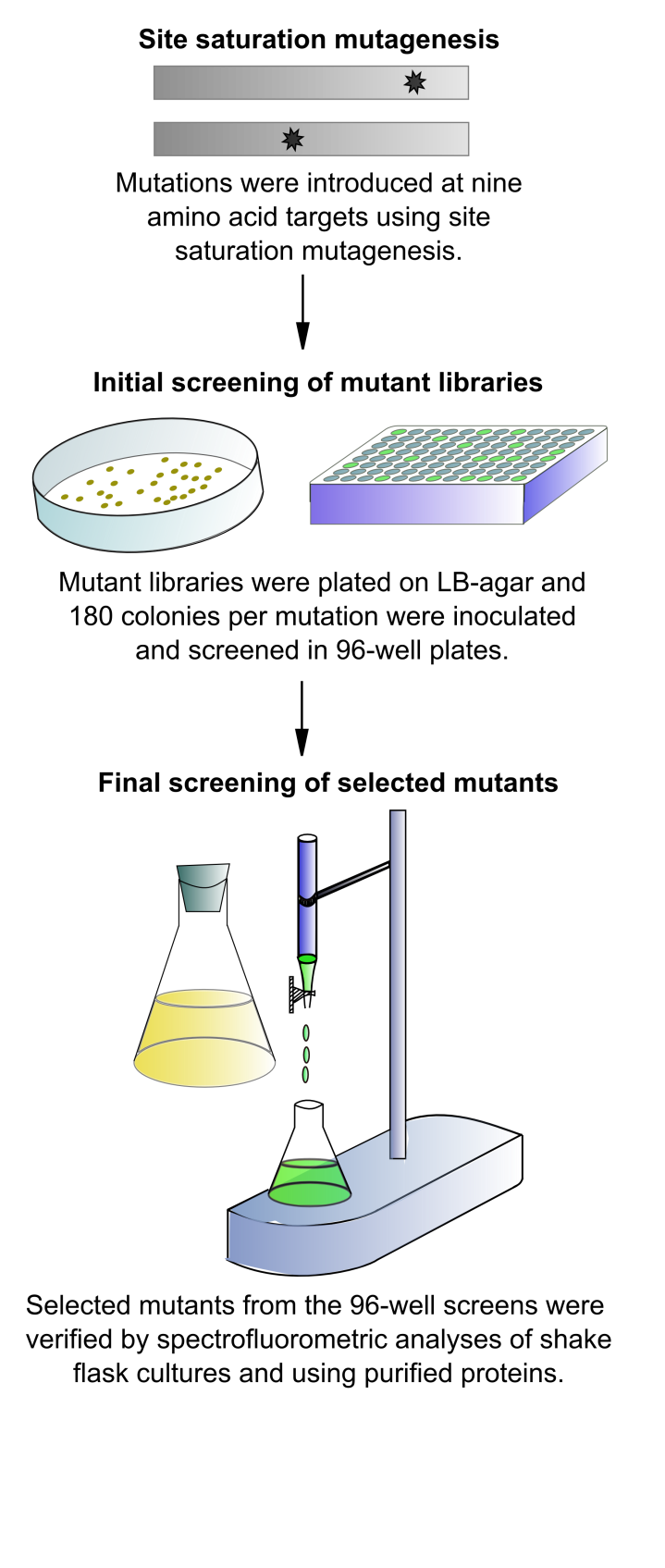


Genes encoding for FbFP are cloned into an IPTG-inducible pQE80L expression vector, followed by site saturation mutagenesis to introduce mutations at specific sites. Mutants are then grown overnight on LB-agar plates and subsequently screened in a 96-well format using spectrofluorometry. Beneficial mutants are identified, selected, and carried forward for growth in shake flask cultures, followed by analysis by fluorescence. Finally, a subset of beneficial mutants are isolated, purified and further analyzed by spectrofluorometry using purified protein preparations.
