# Supplementary material for: Directed evolution of bright mutants of an oxygen-independent flavin-binding fluorescent protein from Pseudomonas putida
Source: J Biol Eng. 2012 Oct 24;6:20. doi: 10.1186/1754-1611-6-20 (PMC3488000; doi:10.1186/1754-1611-6-20)
Supplement: Additional file 5 — Optimizing conditions for laboratory evolution of FbFP. (a) Expression levels of wild type FbFP protein cloned in E. coli cells and expressed from a T5 or a T7 promoter (pQE80L and pET28a(+) vectors respectively) and using three different induction strategies: IPTG induction for 8 hours, 16 hours, and autoinduction. (b) Transformation efficiencies for pQE80L, pET28a(+) and pET*28a(+) expression vectors. pET*28a(+) is a variant of pET28a(+) engineered to express higher levels of the lac repressor, thereby minimizing leaky expression. The pQE80L expression system under IPTG induction outperformed the others in exhibiting higher transformation efficiency and stronger levels of expression. [file 1754-1611-6-20-S5.docx]

**Optimizing conditions for laboratory evolution of FbFP**
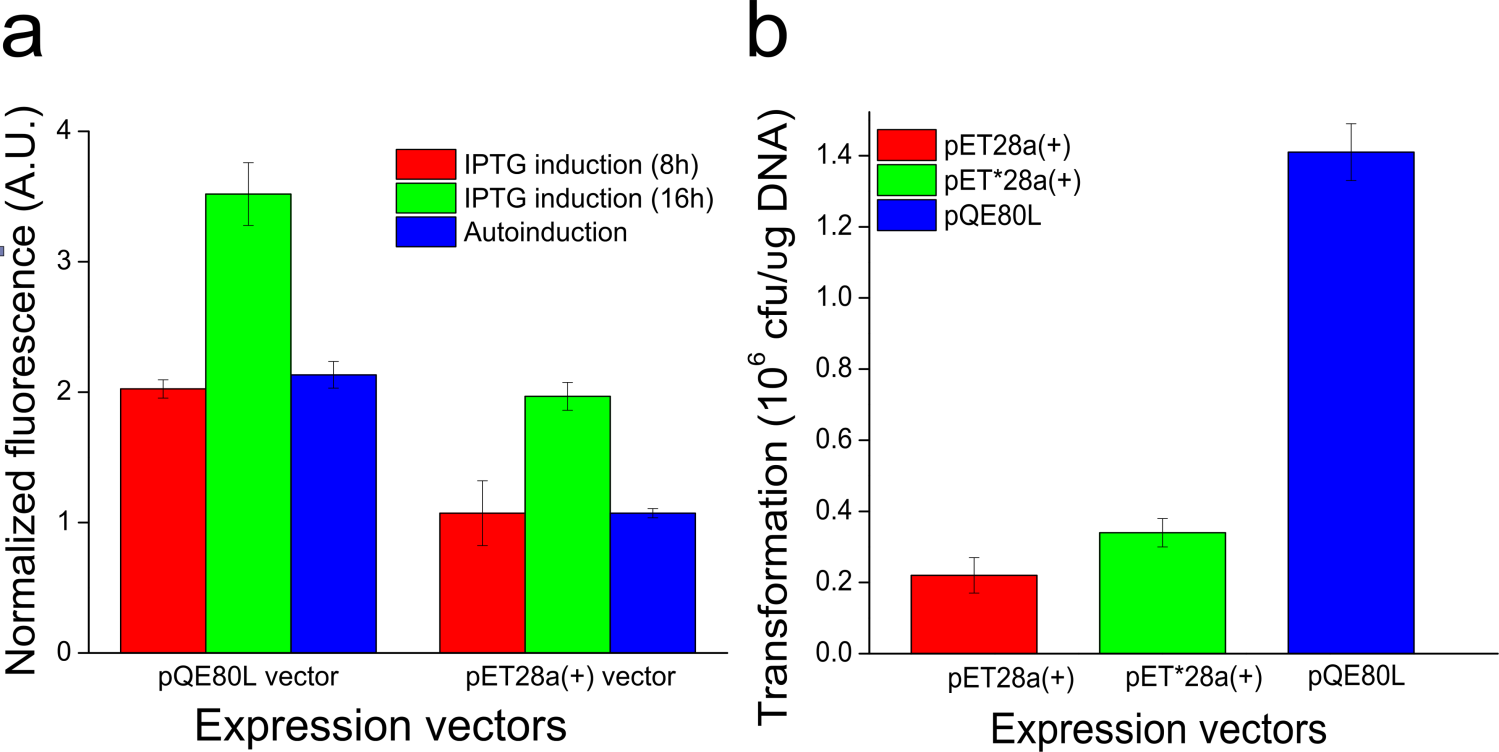


Expression levels of wild type FbFP protein cloned in *E. coli* cells and expressed from a T5 or a T7 promoter (pQE80L and pET28a(+) vectors respectively) and using three different induction strategies: IPTG induction for 8 hours, 16 hours, and auto-induction. **(b)** Transformation efficiencies for pQE80L, pET28a(+) and pET*28a(+) expression vectors. pET*28a(+) is a variant of pET28a(+) engineered to express higher levels of the lac repressor, thereby minimizing leaky expression. The pQE80L expression system under IPTG induction outperformed the others in exhibiting higher transformation efficiency and stronger levels of expression. Auto-induced cultures were grown in ZYP5052 autoinduction media (10 g/L tryptone, 5 g/L yeast extract, 0.5% glycerol, 0.05% glucose and 0.2% lactose). Under auto-induced culture conditions, protein expression is naturally initiated as cells transition from utilizing glucose as the preferred carbon source to lactose concomitant with glucose depletion and diauxic growth. In this way, auto-induction obviates the requirement to precisely identify mid-logarithmic phase of cell cultures for IPTG addition [1].

**References:**

1. Studier FW. 2005. **Protein production by auto-induction in high-density shaking cultures.** Protein Expr Purif 2005, 41:207-234.
