# Supplementary material for: Directed evolution of bright mutants of an oxygen-independent flavin-binding fluorescent protein from Pseudomonas putida
Source: J Biol Eng. 2012 Oct 24;6:20. doi: 10.1186/1754-1611-6-20 (PMC3488000; doi:10.1186/1754-1611-6-20)
Supplement: Additional file 6 — Sequence alignment between FbFP and LOV domains from Chlamydomonas reinhardtii and Arabidopsis thaliana phototropins. Selection of organisms for sequence alignment was motivated by the availability of high quality structures for these LOV proteins. For each alignment, we compared the extent of agreement between amino acids in the chromophore proximal cavity (up to 0.4 nm from FMN) in the homology modeled structure of FbFP and the known crystal structures of other proteins. The chromophore proximal amino acids for each protein are shown in red. Similar amino acids occupying the FMN proximal position in the modeled FbFP structure and the crystal structures of Chlamydomonas and Arabidopsis phototropins (CrPhot and AtPhot respectively) are shown in green. The alignment shows close agreement between the amino acids in the chromophore proximal cavities of our homology model and the existing X-ray diffraction structures of LOV domain proteins from other organisms. [file 1754-1611-6-20-S6.docx]

**Sequence alignment between FbFP and LOV domains from *Chlamydomonas reinhardtii* and *Arabidopsis thaliana*** **phototropins**

**
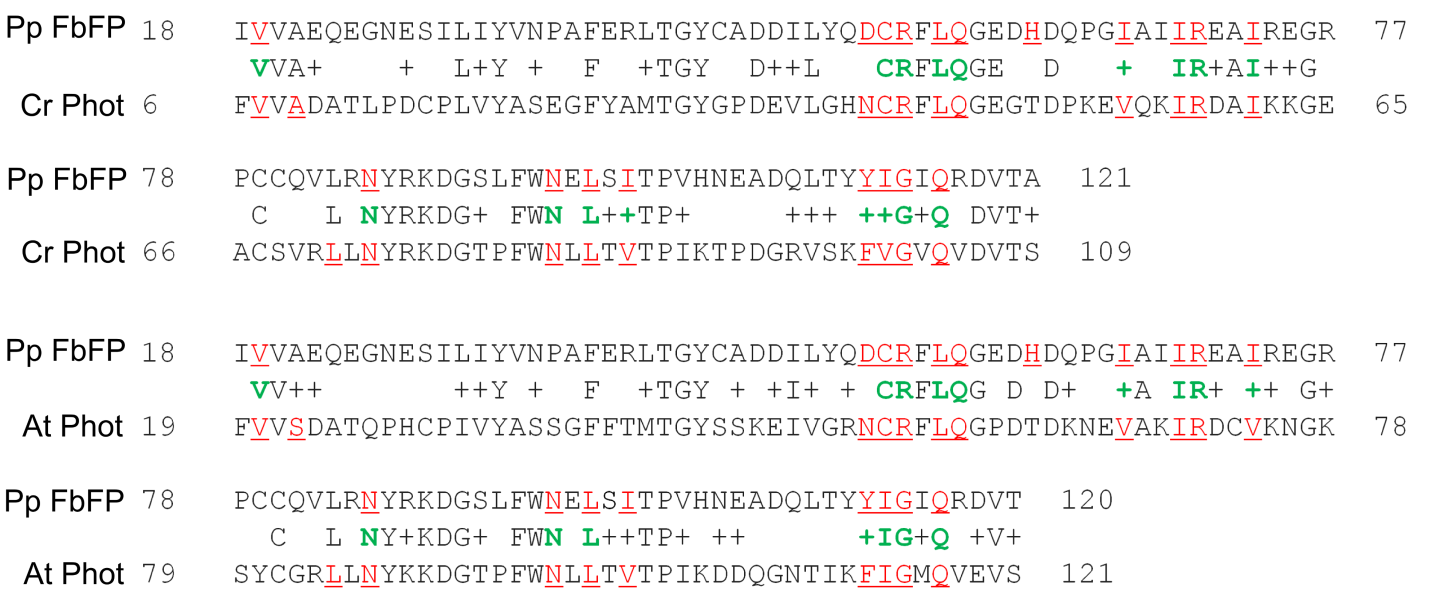
**

Selection of organisms for sequence alignment was motivated by the availability of high quality structures for these LOV proteins. For each alignment, we compared the extent of agreement between amino acids in the chromophore proximal cavity (up to 0.4 nm from FMN) in the homology modeled structure of FbFP and the known crystal structures of other proteins. The chromophore-proximal amino acids for each protein are shown in red. Similar amino acids occupying the FMN proximal position in the modeled FbFP structure and the crystal structures of *Chlamydomonas* and *Arabidopsis* phototropins (CrPhot and AtPhot respectively) are shown in green. The alignment shows close agreement between the amino acids in the chromophore proximal cavities of our homology model and the existing X-ray diffraction structures of LOV domain proteins from other organisms.
