# Supplementary material for: Directed evolution of bright mutants of an oxygen-independent flavin-binding fluorescent protein from Pseudomonas putida
Source: J Biol Eng. 2012 Oct 24;6:20. doi: 10.1186/1754-1611-6-20 (PMC3488000; doi:10.1186/1754-1611-6-20)
Supplement: Additional file 7 — SDS PAGE analysis of purified FbFPs . Purified FbFP fractions migrate as a ~17 kDa band on a 10% polyacrylamide gel. The left-most lane corresponds to the molecular weight ladder. Lanes 1–5 correspond to protein fractions eluted from a two-step chromatographic separation comprising nickel-affinity chromatography and anion-exchange chromatography. [file 1754-1611-6-20-S7.docx]

**SDS PAGE analysis of purified FbFPs**


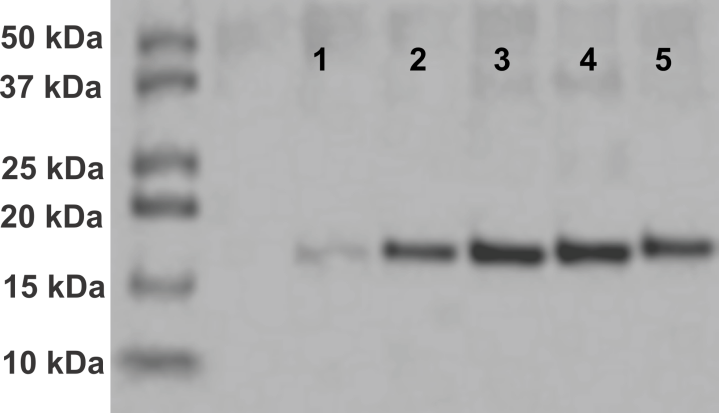


Purified FbFP fractions migrate as a ~17 kDa band on a 10% polyacrylamide gel. The left-most lane corresponds to the molecular weight ladder. Lanes 1-5 correspond to protein fractions eluted from a two-step chromatographic separation comprising nickel-affinity chromatography and anion-exchange chromatography.
